# Snowmaking in a warmer climate: an in-depth analysis of future water demands for the ski resort Andermatt-Sedrun-Disentis (Switzerland) in the twenty-first century

**DOI:** 10.1007/s00484-022-02394-z

**Published:** 2022-12-06

**Authors:** Maria Vorkauf, Robert Steiger, Bruno Abegg, Erika Hiltbrunner

**Affiliations:** 1 Institute of Botany, Department of Environmental Sciences, University of Basel, Schönbeinstrasse 6, 4056 Basel, Switzerland; 2 Faculty of Economics and Statistics, Department of Public Finance, University of Innsbruck, Universitätsstrasse 15, 6020 Innsbruck, Austria; 3 Institute for Systemic Management and Public Governance, University of St. Gallen, Dufourstrasse 40a, 9000 St. Gallen, Switzerland

**Keywords:** Climate change, Scenarios, Ski tourism, Snowmaking, Swiss Alps, Water consumption

## Abstract

Rising air temperatures threaten the snow reliability of ski resorts. Most resorts rely on technical snowmaking to compensate lacking natural snow. But increased water consumption for snowmaking may cause conflicts with other sectors’ water uses such as hydropower production or the hotel industry. We assessed the future snow reliability (likelihood of a continuous 100-day skiing season and of operable Christmas holidays) of the Swiss resort *Andermatt-Sedrun-Disentis* throughout the twenty-first century, where 65% of the area is currently equipped for snowmaking. Our projections are based on the most recent climate change scenarios for Switzerland (CH2018) and the model *SkiSim 2.0* including a snowmaking module. Unabated greenhouse gas emissions (scenario RCP8.5) will cause a lack of natural snow at areas below 1800–2000 m asl by the mid-twenty-first century. Initially, this can be fully compensated by snowmaking, but by the end of the century, the results become more nuanced. While snowmaking can provide a continuous 100-day season throughout the twenty-first century, the economically important Christmas holidays are increasingly at risk under the high-emission scenario in the late twenty-first century. The overall high snow reliability of the resort comes at the cost of an increased water demand. The total water consumption of the resort will rise by 79% by the end of the century (2070–2099 compared to 1981–2010; scenario RCP8.5), implying that new water sources will have to be exploited. Future water management plans at the catchment level, embracing the stakeholders, could help to solve future claims for water in the region.

**Supplementary Information:**

The online version contains supplementary material available at 10.1007/s00484-022-02394-z.

## Introduction

Winter tourism is an important economic sector in mountain regions. Globally, the European Alps are the number one destination for skiing, with 43% of all skier days worldwide. With 24.9 Mio registered skier days in 2018/19, Switzerland ranks as number six in the world (Vanat 2021). In the winter season 2018/19, the Swiss cable cars yielded revenues of 758 Mio CHF (transport only; SBS 2019), underpinning the substantial economic value.

Rising temperatures due to ongoing and future climate change (Rebetez and Reinhard 2008; IPCC 2018) entail severe reductions in the snow cover (Marty 2008; Klein et al. 2016; NCCS 2018; Hock et al. 2019). For the Swiss Alps, winter and spring temperatures are projected to increase by 1.8 K by the end of the twenty-first century if we drastically reduce greenhouse gas emissions, or even up to 3.9 K without any abatement measures (high-emission scenario). Winter precipitation will progressively fall as rain instead of snow and may increase by 12%. However, the projections for the precipitation increase are less clear than for air temperature (NCCS 2018). Winter runoff will increase and the peak runoff will occur earlier because of earlier snowmelt (Haeberli and Weingartner 2020).The operators of ski areas are thus confronted with major challenges for the future. The snow reliability of resorts has often been assessed by means of the 100-day rule (Witmer 1986, for instance used by Abegg et al. 2007; Scott et al. 2008; Steiger and Abegg 2013), stating that a resort requires at least 100 consecutive days with a sufficient snow cover (≥ 30 cm). However, snow reliability does not necessarily result in economic profitability. Another indicator is the Christmas rule introduced by Scott et al. (2008), specifying that the 2 weeks over the Christmas and New Year’s break are a crucial time period for the operators, as these holidays can yield around one quarter of the revenues (Abegg 1996).

The dominant adaptation strategy of operators to cope with climate change and variability is technical snowmaking (OECD 2007; Gonseth and Vielle 2019; Spandre et al. 2019b; Steiger et al. 2019). Currently, the majority of ski slopes in the European Alps are equipped for snowmaking. According to SBS (2021), the area covered with snowmaking in Switzerland massively increased from 14% (2004) to 48% (2014). Today (2020), 53% of all slopes can be snowed-in technically. This is still markedly less than in Italy (90%) and in Austria (70%), but more than in France (37%). The costs for snowmaking, including the water consumption, are substantial. In Switzerland, these amount to 17% of the daily operating expenses (average for resorts with > 25 Mio CHF revenue; SBS 2021).

Surveys among stakeholders in the skiing industry have shown that the operators of ski resorts are very aware of climate change (Abegg et al. 2008). Nevertheless, many do not perceive it as an immediate threat and empathise the high priority of economic competition and short-term weather variability as a major cause for revenue fluctuations (Saarinen and Tervo 2006; Hopkins 2015; Abegg et al. 2017). The adaptation strategy to these more short-term challenges is often also technical snowmaking (Trawöger 2014). A study in Austria highlighted a high confidence in snowmaking facilities, even in low-elevation resorts (Wolfsegger et al. 2008). However, increasing temperatures will reduce the snowmaking potential, as high temperatures and/or high relative humidity inhibit the snow production (Willibald et al. 2021). From 1961 to 2020, the number of hours allowing for snowmaking decreased on average by 26% in Austria, with more pronounced reductions at elevations between 1000 and 1500 m asl (Olefs et al. 2020). Nonetheless, water demand is expected to markedly increase by 50% to 110% across the Alps, according to Steiger et al. (2019). These higher water demands for snowmaking must be put in perspective to water uses in other sectors, such as hydropower production, agriculture, and tourism infrastructures, as well as their future demands under a warmer climate.

The ski resort *Andermatt-Sedrun-Disentis* has recently expanded the ski area with roughly 68 ha of new slopes and with new snowmaking facilities. Such major interventions in the landscape become more and more controversial, especially in times of climate change and a declining demand for ski tickets. Moreover, the short planning horizon of operators does not account for the rising water demand for snowmaking that is very likely under future climatic conditions. Our detailed information about the snowmaking facilities and the snowmaking practices of the operators allow us to present an in-depth analysis of the ski areas future snow reliability throughout the twenty-first century, using the *SkiSim 2.0* model developed by Steiger (2010; based on the *SkiSim 1.0* model by Scott et al. 2003). Based on the RCP (Representative Concentration Pathway) scenarios for Switzerland (NCCS 2018), we simulate the future snow cover in the ski area and assess the snow reliability in terms of the 100-day and the Christmas rule. *SkiSim 2.0* includes a snowmaking model, enabling us to estimate the future water consumption for snowmaking. We expect a strong decline in the natural snow reliability by the mid-twenty-first century that will likely be compensated by snowmaking. We hypothesize that maintaining the resort’s snow reliability will only be feasible at the costs of a strongly enlarged water demand.

## Material and methods

### Ski resort Andermatt-Sedrun-Disentis

**Fig. 1:**
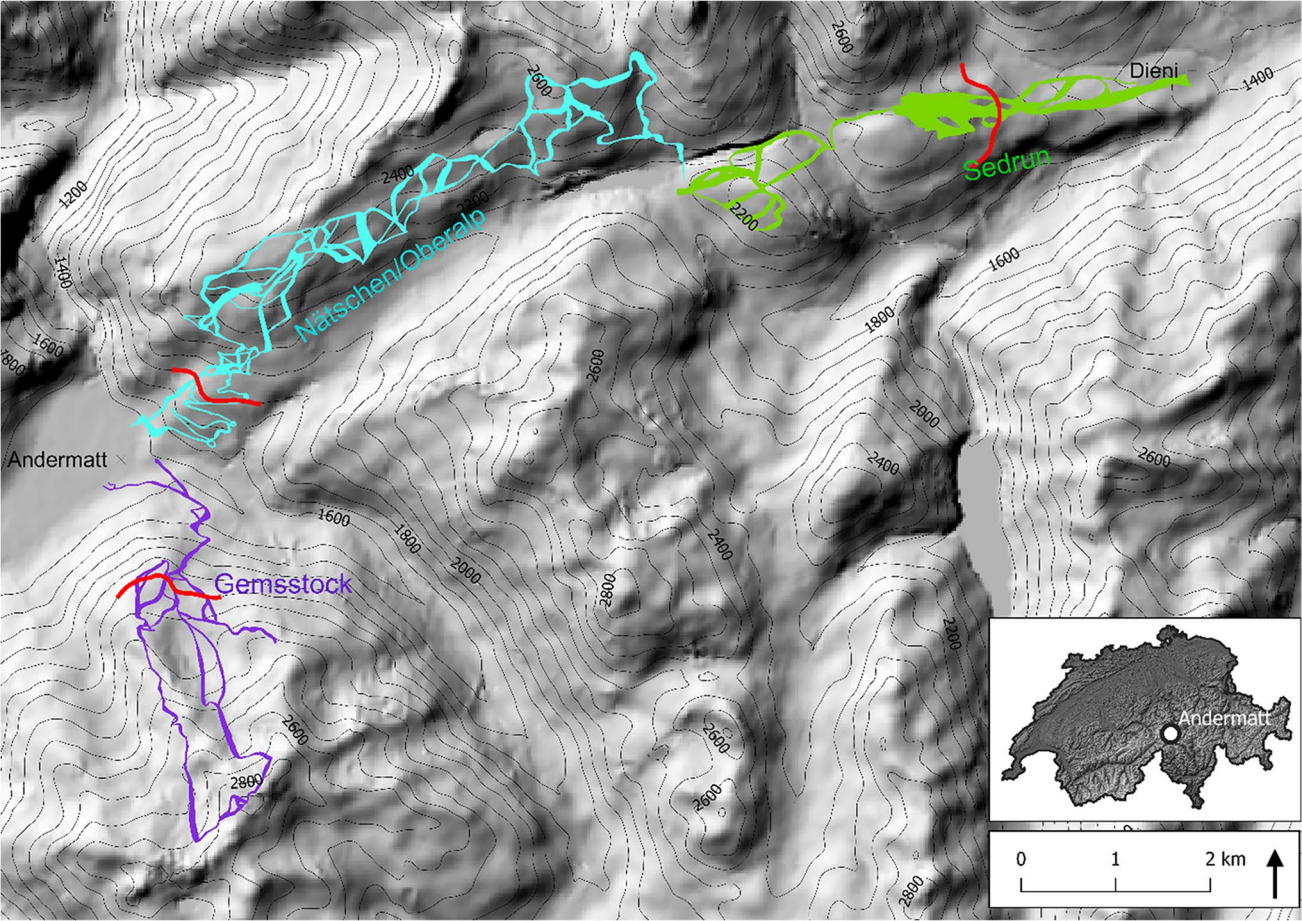
Map with the three ski regions Gemsstock, Nätschen/Oberalp and Sedrun. The red line within each region indicates the critical access elevation, above which skiing is possible even if the lower areas are closed. The miniature map of Switzerland shows the location of Andermatt

The ski resort *Andermatt-Sedrun-Disentis* in the Swiss central Alps has formerly consisted of two separate skiing regions (Gemsstock/Nätschen and Sedrun/Disentis) that were connected by railway from Andermatt to Sedrun/Disentis (Fig. 1). An ambitious project that was launched in 2005 scheduled the expansion of the ski area along with the construction of luxury hotels, penthouse apartments and a golf course. From 2015 to 2018, 130 to 150 Mio CHF were invested to connect the two ski regions with 68 ha of new ski runs, the construction or replacement of 14 ski lifts and a large-scale expansion of the snowmaking facilities. The entire resort *Andermatt-Sedrun-Disentis* comprises around 270 ha of skiing slopes, 175 ha of which are equipped for snowmaking. With the expansion, the operators obtained an additional concession to build a new reservoir lake and to use ground water in Andermatt whenever the water consumption exceeds the current availability (personal communication with former CEO Silvio Schmid).

The highest point of the ski area is on the Gemsstock at 2961 m asl, the lowest point in Andermatt at 1444 m asl. Because of different snowmaking capacities and different water sources, we divided the ski area with approximately 270 ha of ski runs in three regions: (1) Gemsstock, (2) Nätschen/Oberalp, and (3) Sedrun (Fig. 1; see Electronic Supplementary Material [ESM 1] for the official map).

**Fig. 2:**
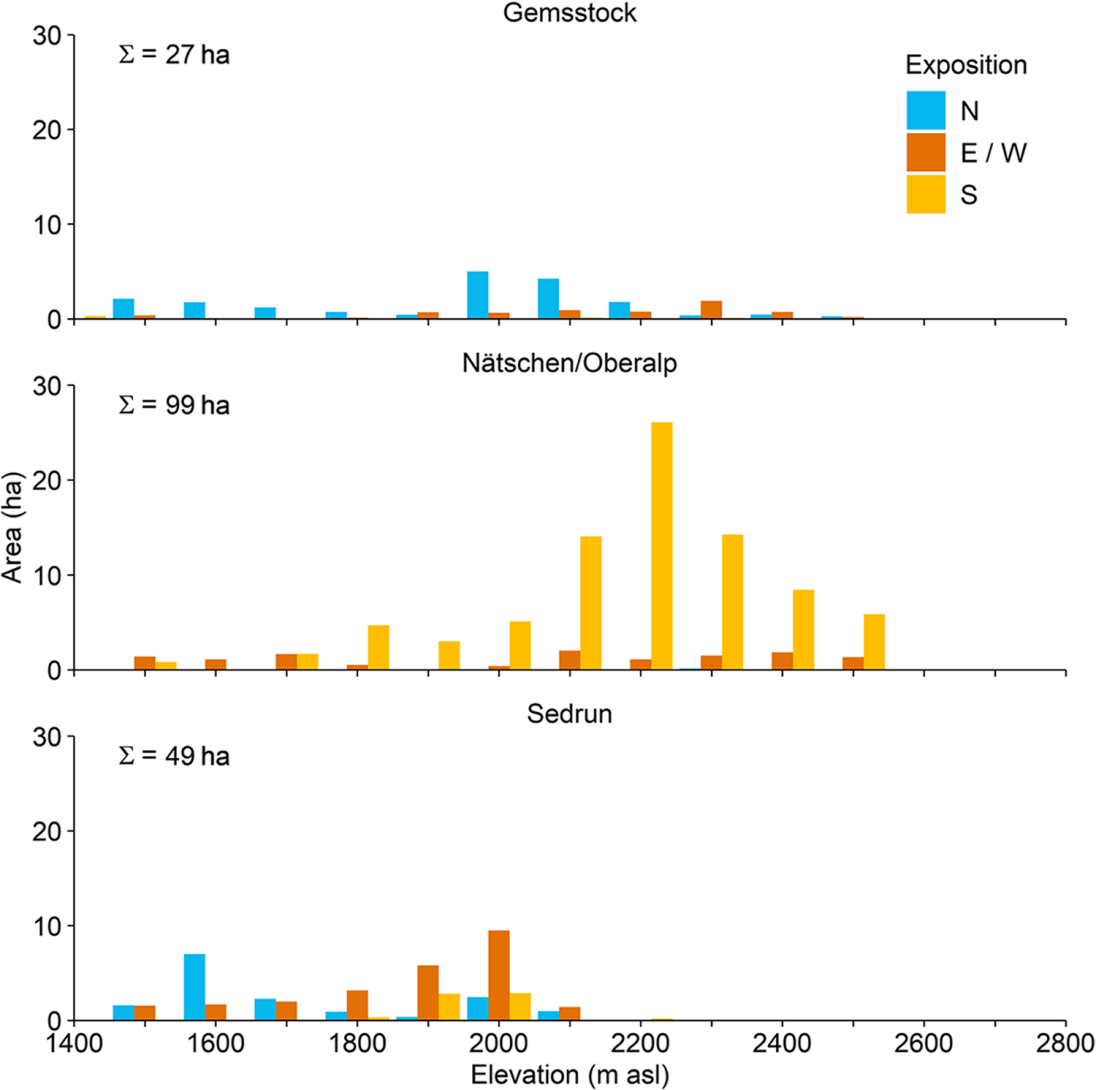
The operative areas for snowmaking along the elevational bands and for the aspects north (N), east or west (E/W; together), and south (S) in the three skiing regions. Currently, around 65% of the skiing slopes can be technically snowed-in

The region Gemsstock is known for freeriding and has mostly northernly exposed ski runs, partly on the small Gurschen and St. Anna firns. The area that is operative for snowmaking (roughly 27 ha, Table 1), is mostly situated below 2100 m asl. The more southernly exposed region of Nätschen/Oberalp (Fig. 2) includes most of the newly built ski runs and chairlifts. Almost the entire region is now equipped with modern snowmaking facilities featuring the highest water pumping rates (Table 1) and with a serviceable area of roughly 99 ha. The highest point of the region is on 2600 m asl. The region Sedrun goes up to 2350 m asl and is the lowest of the three regions. The slopes mainly face towards north or east/west (Fig. 2), and all facilities for snowmaking in this region have existed already before the investments between 2015 and 2018, covering an area of 49 ha for snowmaking. The infrastructure dates back to the 1990ies.

**Table 1:** Snowmaking information for the three regions of *Andermatt-Sedrun-Disentis*, obtained from the operators

Gemsstock	Season: November–May	
Area with snowmaking (% of total)	27 ha (53%)	
Water extraction	Gurschenbach (river)	Mühle (river)
Usual start of snowmaking	Mid-October	November
Usual end of snowmaking	January	January
Pumping rates	60 L s^−1^	100 L s^−1^
Maximum wet bulb temperature	− 1.5 °C	
Nätschen**/**Oberalp	Season: December–April	
Area with snowmaking (% of total)	99 ha (80%)	
Water extraction	Oberalpsee (lake)	
Usual start of snowmaking	Mid-November	
Usual end of snowmaking	January	
Pumping rates	270 L s^−1^	
Maximum wet bulb temperature	− 1.5 °C	
Sedrun	December to April	
Area with snowmaking (% of total)	49 ha (50%)	
Water extraction	Mulinatsch (river)	Val Val (river)
Usual start of snowmaking	November	November
Usual end of snowmaking	January	January
Pumping rates	25 L s^−1^	50 L s^−1^
Maximum wet bulb temperature	− 2 °C

For each of the three regions, we identified a critical access elevation (red line in Fig. 1) based on the skiing infrastructure. The area above these critical access elevations can be reached via cable cars. Hence, the ski regions may remain operable even if the slopes below these elevations have to stay closed. If the slopes above the critical access elevations become unskiable, the region would not be operational anymore. In the Gemsstock region, the critical access elevation is at 2000 m asl, on Nätschen/Gütsch at 1800 m asl, and in Sedrun at 1900 m asl.

### The model SkiSim 2.0

The model *SkiSim 2.0* computes the daily snowpack (in mm water equivalents) considering natural and man-made snow using two modules: (1) a natural snow and (2) a snowmaking module. The natural snow module is a degree day model using daily mean temperature and precipitation as input data. In order to distinguish between snow and rain events and a snow/rain mixture, a lower and an upper daily mean temperature threshold is calibrated based on daily snow fall data (< 1 °C: snow, > 3 °C: rain, between: snow/rain mix).

The daily melt is estimated based on degree days (daily mean temperature > 0 °C). The so-called degree day factor, which describes the melt that occurs per degree day is also fitted during the model calibration process with the number of snow days (snow depth ≥ 1 cm) per season (ESM 2). The number of snow days was slightly overestimated by the model (1.7% in Andermatt and 3.1% in Sedrun; Table 2). For further details on the natural snow module, refer to Steiger (2010).

**Table 2:** The observed *vs*. modelled number of days with natural snow cover (≥ 1 cm) for the calibration (1981–1987) and for the validation period (1988–2010) at the stations Andermatt and Sedrun. *R*^2^ is the coefficient of determination for the respective period

	Days with snow cover		
	observed	modelled	R^2^
Andermatt			
calibration	167.7	166.9	0.89
validation	160.3	163.0	0.83
Sedrun			
calibration	159.1	159.9	0.85
validation	150.3	155.0	0.73

The years 1981 to 1987 were used for model calibration and 1988 to 2010 for model evaluation (both periods together denote the reference period). In this application, we used separate degree-day factors for three aspect classes: − 25% for north exposed ski slopes, + 25% for south exposed slopes and an unchanged calibrated degree-day factor for east and west-facing slopes.

The weather stations Andermatt (1442 m asl; ESM 3) and Sedrun (1429 m asl) were used for the input data, Andermatt for the regions Gemssock and Nätschen/Oberalp, and Sedrun for the region Sedrun. Temperature and precipitation are extrapolated from the elevation of these weather stations to the elevation range of the ski areas in 100 m bands. We used a region-specific lapse rate of the air temperature between Sedrun/Gütsch (2287 m asl) and Andermatt/Gütsch, respectively, that was fitted during model calibration. Separate lapse rates were calculated for each month of the year, and for dry (< 1 mm precipitation) and wet days (≥ 1 mm), respectively. For the precipitation, we assumed a constant 3% increase per 100 m of elevation (Steiger 2010).

The snowmaking module takes into account that the operators of the ski area start to produce snow at certain dates (see Table 1), provided temperatures are low enough. For comparability with other *SkiSim* studies (e.g., Steiger and Scott 2020) we used − 2 °C air temperature as threshold for snowmaking. Note that this threshold is rather conservative given the wet-bulb temperature threshold provided by the ski area operators (Table 1). For instance, a wet-bulb temperature of − 2 °C corresponds to − 1 °C air temperature at 80% humidity, while at 100% humidity no evaporative cooling occurs. Snow is produced until the base layer is 30 cm thick (corresponding to a snow water equivalent of 120 mm at a snow density of 400 kg m^−3^). This is the so-called base-layer snowmaking, which is required for skiing. Thereafter, more snow is produced to sustain skiing until the end of the scheduled season. In the model, the snow production is calculated hourly, under the assumption of interpolated daily minimum and maximum temperatures. Refer to Steiger (2010) for a detailed description of the snowmaking module.

We ran the model for each of the three regions (Gemsstock, Nätschen/Oberalp, Sedrun) separately, each divided into elevational bands of 100 m.

We computed the water consumption for a hydrological year that includes the full skiing season (year *y* runs from Sept 1^st^ *y-1* to Aug 31^st^ *y*). Based on the daily snowpack, we determined the probability of a continuous snow cover for 100 days in a row (100-day rule) and of a continuous snowpack over Christmas and New Year (Christmas-rule; defined as Dec 22^nd^ to Jan 4^th^). As suggested by Abegg et al. (2021), the selection of the snow reliability indicators was done in close co-operation with the ski area operators. Snow reliability and *high* snow reliability are given when the 100-day rule is fulfilled in 70% and 90% of the winters, respectively, as ski areas are expected to be able to withstand single years with less favorable conditions. We defined the snow reliability of the Christmas-rule in the same way. To achieve results that are representative for the entire regions, we calculated area-weighted means of the probabilities, accounting for the area equipped for snowmaking in the elevational bands and in the aspect classes, as in Steiger and Stötter 2013 (and similar to François et al. 2014, who weighted by ski lift power). The probabilities of each simulation were calculated based on the number of years in the 30-year time period when the 100-day rule or the Christmas rule were fulfilled. If not indicated differently, all reported probabilities refer to the median of all simulations in a RCP scenario (RCP2.6, RCP4.5 and RCP8.5, see below) for a given time period during the twenty-first century (three time periods, see below). When results are visualized for single aspects they always refer to the east/west aspect (north and south aspect presented in the ESM).

The information about technical issues (area equipped for snowmaking, pumping rates, allowed water extraction) and about snowmaking practices (adopted wet-bulb temperatures, start dates, see Table 1) was obtained from *Andermatt-Sedrun-Disentis* directly (formerly *SkiArena Andermatt Sedrun*).

### Climate change scenarios and data availability

The CH2018 climate change scenarios were produced for single weather stations (see ESM 3 for the station Andermatt) as well as for a 2 × 2-km grid over whole Switzerland (NCCS 2018). There are three RCP scenarios with a total of 68 simulations: RCP2.6 (greenhouse gas emission stop with warming of less than 2 K compared to pre-industrial times; 12 simulations), RCP4.5 (emission stop in second half of the twenty-first century, warming > 2 K; 25 simulations), and RCP8.5 (high-emission scenario without emission stop; 31 simulations). These scenarios include daily simulations of temperature and precipitation until the year 2099. Here, we present the results for three time periods: 2020–2049 (early century), 2045–2074 (mid-century), and 2070–2099 (end of century). We refer to the 30-year periods of the RCP scenarios to get an estimate of the future conditions under the three scenarios. However, single extreme years may differ significantly from these estimates (for instance, “avalanche winter” 1999, ESM 3).

For the weather station in Andermatt, the scenarios for the twenty-first century do not include any simulations for the minimum air temperature. We therefore used the gridded scenarios and extracted the input data for *SkiSim 2.0* (minimum and maximum air temperature, precipitation) from the 2 × 2-km grid cell containing Andermatt. To account for the elevational difference between the grid cell and the weather station, we applied the temperature and precipitation lapse rates of the model.

### Water consumption

We modelled the water consumption of the ski area for the reference period of the climate change scenarios (1981 to 2010). Theoretically, these numbers could then be compared to the actual water usage of that period. However, actual water usage is only available for the winters 2002–2017 (Sedrun) and 2014–2017 (Gemsstock). While these numbers refer to the snowmaking facilities of that time, the modelled water consumption is based on the full expansion of the facilities as in 2018. Thus, we could only carry out a plausibility check on the modelled numbers of the water consumption (see result section).

## Results

### Future snow reliability

**Fig. 3:**
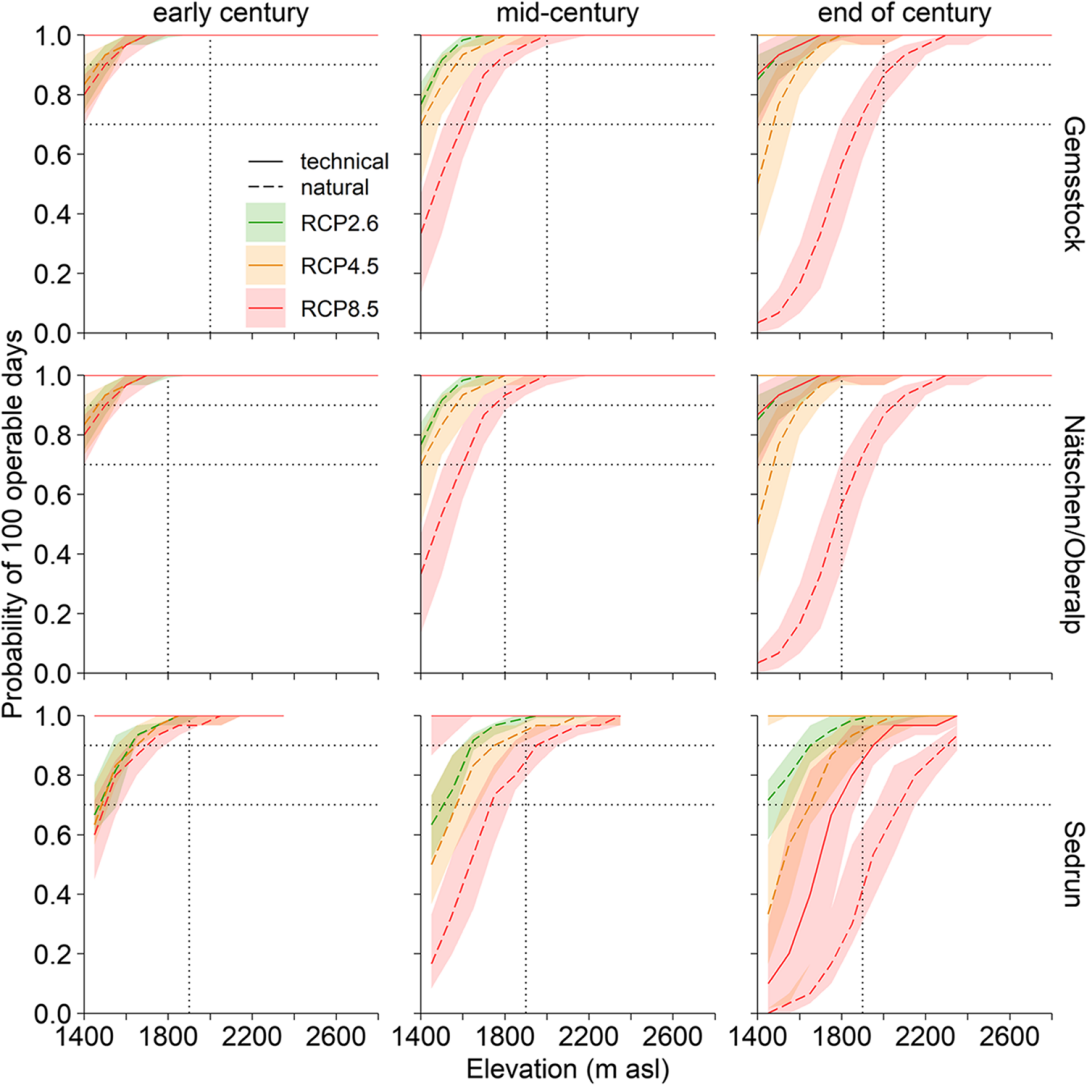
The probability of 100 consecutive days that are operable for skiing on natural snow (dashed line) and with technical snow (solid line) for the three regions Gemsstock, Nätschen/Oberalp, Sedrun under the three RCP scenarios and for three time periods of the twenty-first century. The lines represent the median of all simulations per RCP scenario and 50% of the simulations lie in the shaded ribbon. The horizontal lines indicate the snow reliability at 70% and 90%, the vertical lines refer to the critical access elevation. All results refer to an east/west aspect, north and south exposures are in ESM 4 and 5. At a probability of 1, the lines of the three scenarios overlap

In terms of the 100-day rule and of our 70% threshold, the ski area is naturally snow reliable throughout the early twenty-first century, especially above the critical access elevations (Fig. 3; see ESM 4 and 5 for north and south exposure). Under RCP8.5, the snow reliability below 1700 m asl starts to drop below 70% without any technical snow, but it can be maintained high with snowmaking (ESM 6). It continues to decrease towards the end of the twenty-first century, but mainly under RCP8.5. While snowmaking will compensate for the lack of natural snow in the regions of Gemsstock and Nätschen/Oberalp, in Sedrun, this will not be feasible at elevations below 1800 m asl (Fig. 3; east/west exposure). Compared to the east/west aspect, the snow reliability is lower on southernly exposed slopes, especially below the critical access elevation, where the snow reliability is generally lower (natural snow: 13–23%; technical snow: 0–13%; below the critical access elevation under RCP8.5 by end of century) and it is higher on northernly exposed slopes (natural snow: 7–23%, technical snow: 0–10%; ESM 5).

**Fig. 4:**
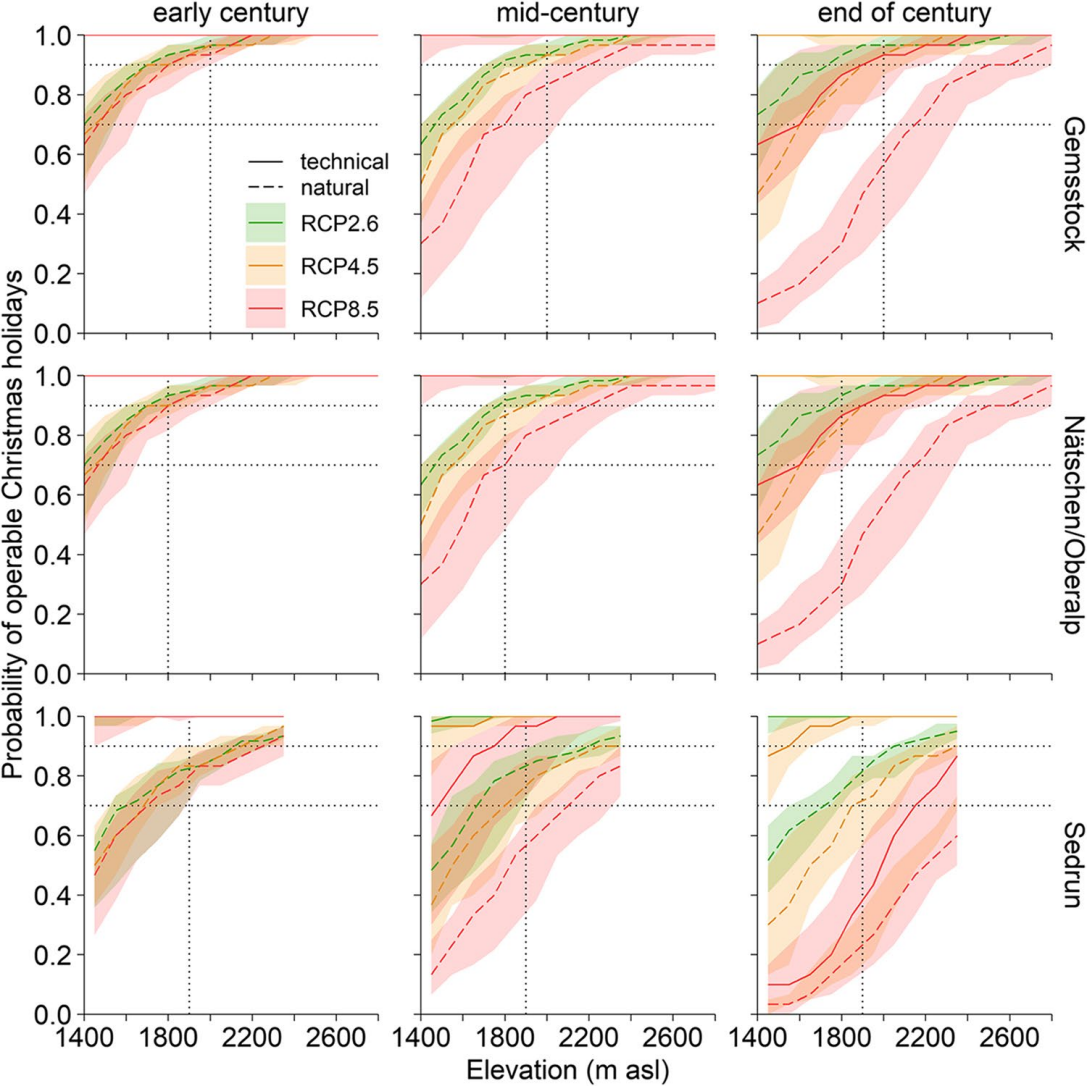
The probability that the resort is operable during the Christmas holidays on natural snow only (dashed line) and with technical snow (solid line) for the three regions Gems-stock, Nätschen/Oberalp, Sedrun under the three RCP scenarios and for three time periods of the twenty-first century. See legend of Fig. 3 for a detailed description. All results refer to an east/west aspect, north and south exposures are in ESM 7 and 8

**Fig. 5:**
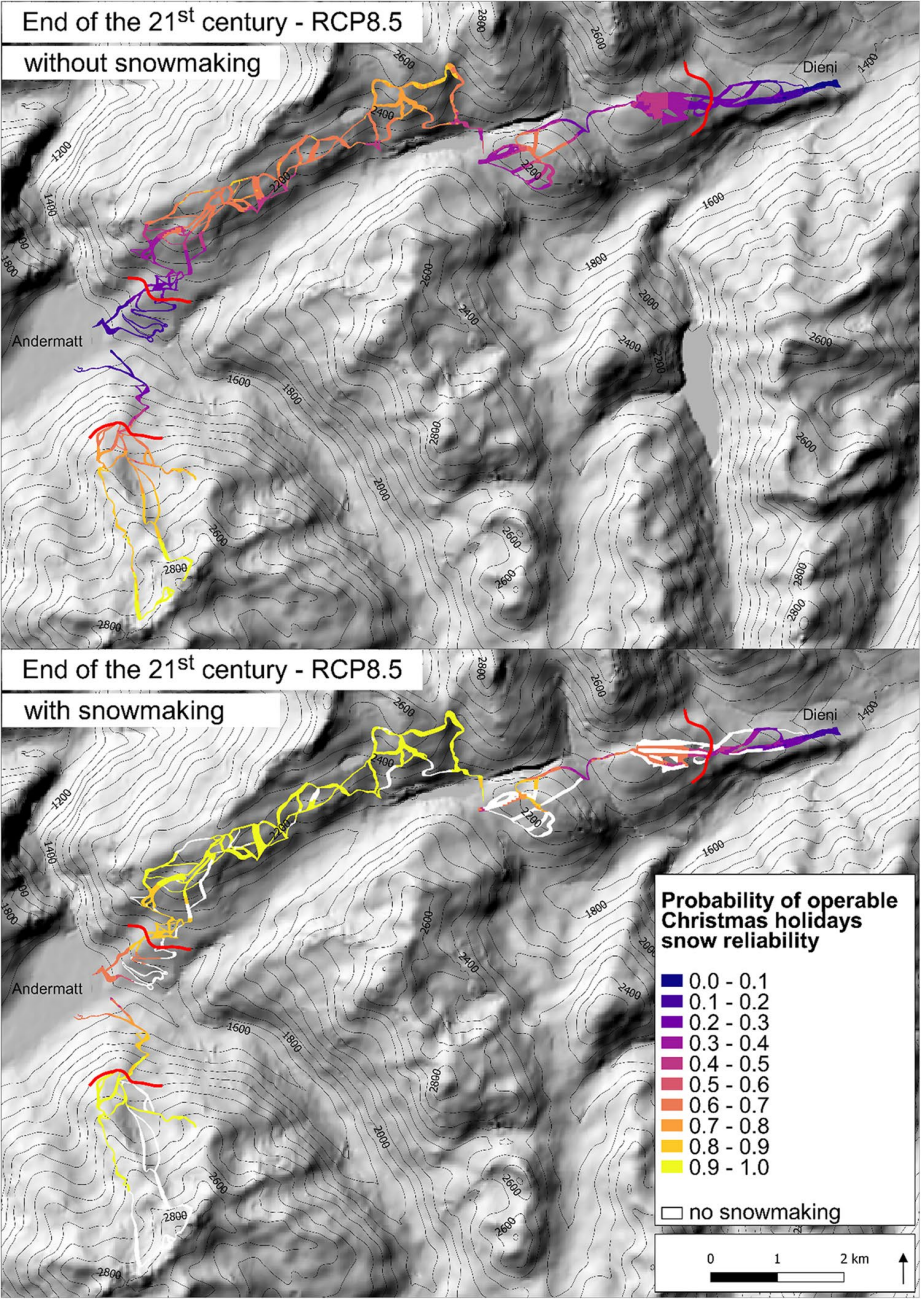
The probability of operable Christmas holidays with and without snowmaking at the end of the century under RCP8.5. Areas depicted in white are not serviceable for snowmaking and the red line is the critical access elevation

The natural snow reliability over the Christmas holidays is generally lower than for an operable season of 100 days (Fig. 4; see ESM 7 and 8 for north and south exposure). Snowmaking will mostly allow skiing over the holidays. However, under RCP8.5 Christmas skiing becomes increasingly unlikely by the end of the century (RCP8.5; Fig. 5). The influence of southernly exposed slopes on the snow reliability is somewhat smaller over the Christmas holidays than for the 100-day rule (natural snow: 3–13%, technical snow. 3–7%; below critical access elevation under RCP8.5, end of century; ESM 8). This smaller impact of the exposure is most likely due to lower temperatures in December and January.

### Gemsstock

The natural snow reliability of the Gemsstock region is generally high throughout the whole twenty-first century (100-day rule; Fig. 3), particularly above the critical access elevation of 2000 m asl. By the end of the century, natural snow will not suffice to sustain a continuous skiing season of 100 days. But this concerns the lower areas under the RCP8.5 scenario only and may be compensated with technical snowmaking (Table 3).

**Table 3:**
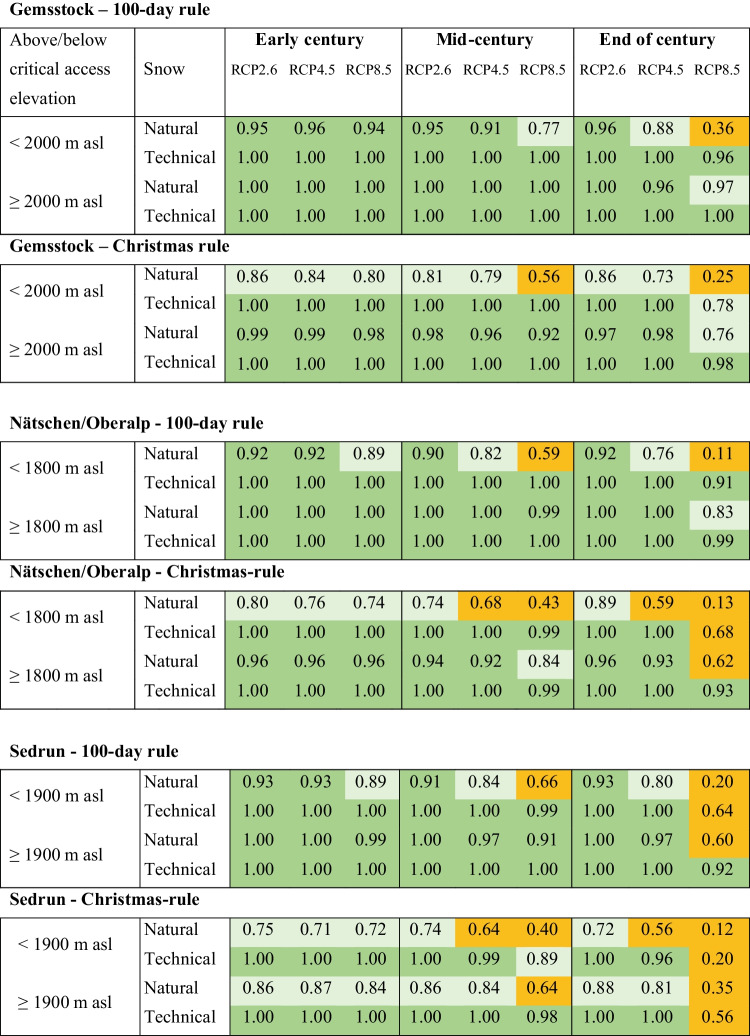
The median likelihood (area-weighted) for fulfilling the 100-day and the Christmas rule below and above the critical access elevation, and under three RCP scenarios (RCP2.6, RCP4.5, RCP8.5) for three time periods on natural snow and with snowmaking. Dark green: highly snow reliable, light green: snow reliable, orange: not snow reliable

The Christmas holidays are snow reliable at the beginning of the century, but the snow reliability gradually decreases towards the end of the twenty-first century (Fig. 4). Under RCP8.5, the low-elevation areas become snow scarce by the mid-century already (enough natural snow in 56% of the winters only). But with snowmaking, the whole Gemsstock region remains snow reliable until the end of the century (Table 3; Fig. 5 and ESM 6).

### Nätschen/Oberalp

Both the natural and technical snow reliability of the region Nätschen/Oberalp are similarly high as in the Gemsstock region (100-day rule; Fig. 3). However, the critical access elevation is at 1800 m asl, above where the slopes can be skied unrestrictedly is 200 m lower than on the Gemsstock. Accordingly, under RCP8.5, the low-elevation areas are not naturally snow reliable anymore by the mid-century already. By the end of the century, natural snow is projected to suffice for a 100-day skiing season in one out of ten winters only (Table 3). Because of the widespread snowmaking facilities, the region will generally remain snow reliable throughout the whole twenty-first century, even under RCP8.5.

However, the Christmas holidays will likely become increasingly snow scarce (Table 3). Below the critical access elevation, producing a base layer of technical snow for Christmas skiing may only be possible in 68% of the winters (RCP8.5).

### Sedrun

Even though Sedrun is the least snow reliable region (Fig. 3), the 100-day rule will still be fulfilled throughout most of the twenty-first century. However, from the mid-century on, the natural snow reliability occurs in 66% of the winters below the critical access elevation of 1900 m asl (RCP8.5). With technical snow it can be maintained very high, fulfilling the 100-day rule in 99% of the winters. By the end of the century, the natural snow reliability at low elevations will only be given every fifth winter, and high temperatures will render sufficient snowmaking impossible (only feasible in 64% of the winters; Table 3). Above the critical access elevation of 1900 m asl, snowmaking maintains the area snow reliable even under RCP8.5 at the end of the century.

The situation during the Christmas holidays is projected to be considerably worse in Sedrun than in the other two regions Gemsstock and Nätschen/Oberalp (Fig. 4). By the mid-century, the natural snow reliability at lower elevations will decrease drastically (RCP4.5 and RCP8.5), even above the critical access elevation of 1900 m asl (RCP8.5; Table 3). The lack of natural snow can be compensated by snowmaking, but under RCP8.5, the region will not be snow reliable anymore, not even with snowmaking above the critical access elevation (Fig. 5, Table 3).

## Water consumption

### Water consumption in the reference period

The mean water consumption in the Gemsstock region between 2014 and 2016 was 48 × 10^3^ m^3^ season^−1^, whereas in the snow scarce winter of 2017 it was 150 × 10^3^ m^3^ season^−1^; thus, it more than tripled. In the region Sedrun, the mean water consumption between 2006 and 2016 was 114 × 10^3^ m^3^ and in the winter 2017, it increased by ca. 70% to 195 × 10^3^ m^3^ season^−1^. For the region Nätschen/Oberalp, the facilities are simply too new to obtain water consumption data of the past winters (running since winter 2019). Because the model assumes a fully expanded ski area, the water consumption of the past years may mainly serve as a plausibility check for the model outcomes.

The modelled baseline water consumption of the three regions of the ski area (reference period 1981–2010) is estimated 46.8 × 10^3^ m^3^ season^−1^ for Gemsstock (17% of the total 301.5 × 10^3^ m^3^ season^−1^; 3% lower than observations 2014–2016), 172.3 × 10^3^ m^3^ season^−1^ for Nätschen/Oberalp (57%), and 82.4 × 10^3^ m^3^ season^−1^ for Sedrun (26%), respectively. When we used the temperature and precipitation of the reference period in Sedrun (instead of the RCP climate change scenarios), the modelled water consumption for the reference period was 99.0 × 10^3^ m^3^ season^−1^ (13% lower than observations 2006–2016).

Our model did not include any “water losses” (see below) and assumed that the operators only produced as much technical snow as required to guarantee a minimum snow depth of 30 cm until the scheduled season ends. However, operators often produce more snow than assumed in our model, as the course of the season is still unknown when the snow is produced (mainly between October/November and January). Thus, our modelled water consumption is rather conservative, and it is therefore likely that our future projections are underestimated.

### Water consumption in the twenty-first century

The total water consumption at the end of the century will increase by 4% (RCP2.6), 16% (RCP4.5), or even 79% (RCP8.5) compared to the baseline, respectively. Below the critical access elevations (1800–2000 m asl), the relative increase in the water consumption will be much higher: 15% (RCP2.6), 47% (RCP4.5), and 195% (RCP8.5; reference 82.1 × 10^3^ m^3^ season^−1^). Above the critical access elevation, increases in water consumption will only amount to 0% (RCP2.6), 3% (RCP4.5), and 35% (RCP8.5) by the end of the century (reference of 219.5 × 10^3^ m^3^ season^−1^).

Hypothetically, the operators could decide to fully abandon snowmaking below the critical access elevations, and only operate the higher areas. This would theoretically diminish the total water consumption of the ski area compared to the reference period, even at the end of the twenty-first century (RCP2.6: − 28%, RCP4.5: − 25%, RCP8.5: − 2%).

If the concentration of greenhouse gas emissions were to stay at today’s levels thanks to successfully applied abatement measures (RCP2.6), the total water consumption of the ski area would only be 4% higher than during the reference period by the end of the twenty-first century. Thus, in the following, we report the results of the RCP4.5 and the RCP8.5 scenarios for each region.

### Gemsstock

**Fig. 6:**
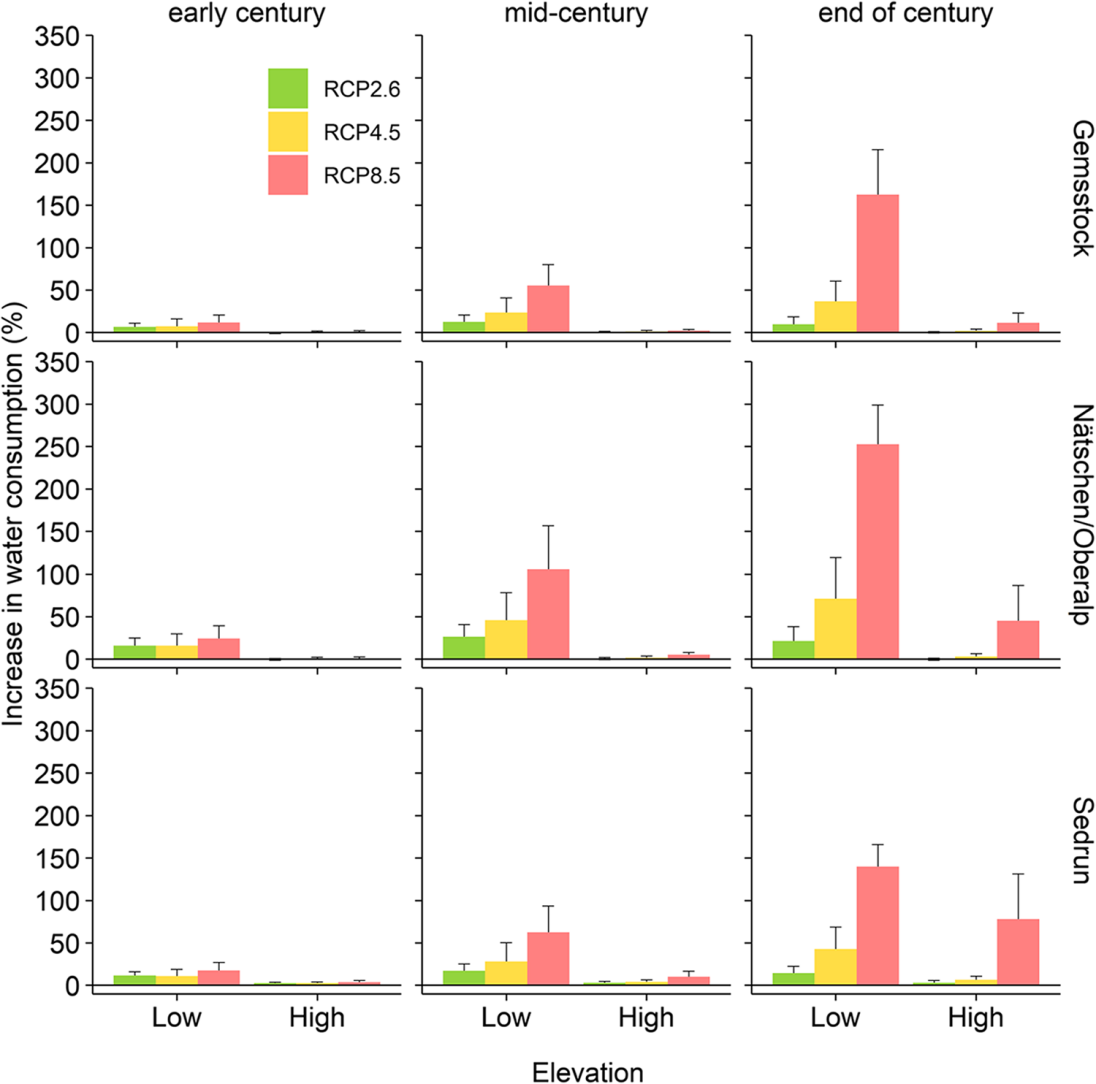
The increase in water consumption for the three regions compared to the baseline (reference 1981–2010; modelled with full expanded skiing resort). High and low elevations are above and below the critical access elevation, respectively

On the Gemsstock, the modelled water consumption for the reference period was 29.5 × 10^3^ m^3^ season^−1^ for areas above the critical access elevation of 2000 m asl, and 17.3 × 10^3^ m^3^ season^−1^ for the lower areas. In line with the high snow reliability throughout the twenty-first century, there is practically no increase in the water consumption above 2000 m asl (+ 7% by the end of the century under RCP8.5). Below the critical access elevation, including the runs to the valley bottom, the water demand in the mid-century will rise by 22% and 51% under RCP4.5 and RCP8.5, respectively, and by 35% (RCP4.5) and 162% (RCP8.5) by the end of the century (Fig. 6).

### Nätschen/Oberalp

Above the critical access elevation of 1800 m asl, the yearly water consumption was 146.4 × 10^3^ m^3^ season^−1^, and 25.9 × 10^3^ m^3^ season^−1^ for the lower situated areas during the reference period. In this region, only 9% of the area equipped for snowmaking lies below 1800 m asl (Fig. 2). As on the Gemsstock, the increase of water consumption above the critical access elevation is moderate (0–5%), except under RCP8.5 at the end of the century with 28%. However, below 1800 m asl, our model projects massive increases in water consumption. Under RCP4.5, these will be 13% (early century), 40% (mid-century), and 61% (end of century), while under RCP8.5, they will even be 24%, 101%, and 271% (Fig. 6).

### Sedrun

In the region Sedrun, the modelled yearly water consumption was 43.6 × 10^3^ m^3^ season^−1^ for elevations above 1900 m asl (critical access elevation), and 38.9 × 10^3^ m^3^ season^−1^ for lower elevations during the reference period. Similar to the other two regions, increases in water consumption will range between 2 and 9%, but under RCP8.5, we project a rise of 78% by the end of the century.

Below 1900 m asl, the water use under RCP4.5 will go up by 39% by the end of the century. Under RCP8.5, the additional water required for snowmaking will increase by 53% in the mid-century, and by 142% by the end of the century (Fig. 6).

## Discussion

### Snow reliability

Our in-depth analysis of *Andermatt-Sedrun-Disentis* shows that the ski area remains snow reliable (consecutive 100-day season) in the twenty-first century, provided that snowmaking will be intensified. Fortunately for the operators, the resort as a whole has multiple entry points that are accessible even when lower parts of the ski area might not be skiable anymore. At high elevations, the entire ski area will be fully operational for at least 100 consecutive days. About three quarters of the total ski area lie above 2000 m asl, and this is also where the majority of the new lifts, slopes and ski runs were constructed (2015–2018). Ultimately, only the snow reliability on the valley runs cannot be maintained. The upper parts of the ski areas, including the newly built slopes, are projected to be operable until the end of the twenty-first century.

Due to future climatic conditions and high investment costs for modern snowmaking facilities (Abegg et al. 2008), there will be a diminishing number of operable ski areas in the future worldwide (e.g., Fang et al. 2019; Spandre et al. 2019b; Steiger et al. 2019; Scott et al. 2020). As the snow scarce winters in the late 1980s showed, high-elevation ski areas can benefit from increased visitor numbers when ski areas at lower elevations close (Koenig and Abegg 1997; Steiger et al. 2019). Accordingly, we assume that in the mid-term, *Andermatt-Sedrun-Disentis* may even profit from the shutting down of other ski areas.

In contrast to the minimal season length of 100 consecutive days, the resort’s situation over the Christmas holidays (Christmas rule) is much less stable throughout the twenty-first century. Unreliable snow conditions for ski areas during Christmas holidays are projected to emerge globally (Berghammer and Schmude 2014; Steiger et al. 2019; Steiger and Scott 2020), as for instance in 2017, when the onset of snow in Andermatt was on January 3^rd^. In *Andermatt-Sedrun-Disentis*, this mainly affects the region Sedrun, where snowmaking will reach its limits by the end of the century. In a comprehensive analysis of 34 ski areas in the canton of Grisons (eastern CH), Abegg et al. (2015) used the same 70% threshold for the 100-day and the Christmas rule for assessing the snow reliability and highlighted that only 15% of the ski areas would be naturally snow reliable by the end of the century. Snowmaking could increase the share of snow reliable ski areas to 56%, but the required snow production would rise by more than 100%. Sedrun was one of the analysed ski areas and they projected that with snowmaking, the ski area would still be snow reliable by the end of the twenty-first century. Our results with a higher spatial resolution and with the latest version of the Swiss climate change scenarios revealed that the ski area will only be partially snow reliable, fulfilling the 100-day rule, but not the Christmas rule anymore.

The low snow reliability in the region Sedrun is partly due to older snowmaking facilities compared to Gemsstock and Nätschen/Oberalp. The technical snow reliability could be increased by renewing these old facilities and thereby allowing for higher water pumping rates (pumping rates at Nätschen/Oberalp are between five to ten times higher than those in Sedrun). This is underpinned by the very snow scarce winter of 2017. While the water consumption in the Gemsstock region tripled, in the region of Sedrun there was an increase of roughly 70% only. It is likely that the older snowmaking facilities restricted the production of technical snow. This reinforces a study across six Norwegian ski resorts, where lower snowmaking capacities were related to a higher vulnerability over the Christmas holidays (Dannevig et al. 2021). However, the natural snow reliability was also lower in Sedrun, therefore, the lower overall snow reliability cannot be attributed to the older snowmaking facilities alone. Generally, there has been a rapid technical development of such facilities, as evidenced by the high pumping rates of the new installations in Nätschen/Oberalp (270 L s^−1^). Such a high performance, in combination with sufficient water supply, is considered crucial for the snow reliability in snow scarce years. Even though further gains in the efficiency of snowmaking facilities are likely, the technology itself is also bound to physical limitations (wet-bulb temperature). Hence, unsuitable climatic conditions as they are often observed at the beginning of the season and/or at low elevations, substantially reduce the potential benefit of snowmaking facilities (Berard-Chenu et al. 2022).

### Water consumption

Generally, the water consumption for snowmaking in the European Alps is estimated to increase between 50 and 110% (by 2050; Steiger et al. 2019). A rising demand for technical snow causes higher costs for the water consumption, but also increased operating costs and additional investments in snowmaking facilities. We project that in an average winter at the end of the century, the entire resort *Andermatt-Sedrun-Disentis* will require 79% more water for snowmaking (RCP8.5; roughly an increase from 300 × 10^3^ m^3^ season^−1^ to 540 × 10^3^ m^3^ season^−1^). When we take the 730 L day^−1^ water consumption of a typical 4-person household as a reference (Abwasser Uri 2019), the water consumption for snowmaking would increase from approximately 1130 to 2020 households. Further investment in the snowmaking capacity would intensify the water consumption beyond our model results. In the French Alps, the water consumption by the end of the century could even increase by the ninefold, if the area for snowmaking would be increased to 100% (Spandre et al. 2019a).

The model *SkiSim 2.0* does not account for water losses through wind drift, sublimation, and evaporation, which may lead to the underestimation of the water consumption during the process of snowmaking. Grünewald and Wolfsperger (2019) highlighted that water losses ranged between 7 and 35%, depending on weather conditions. In their field tests, water losses augmented by 2.8% per 1 K increase in air temperature. Future conditions for snowmaking will become increasingly unfavourable and hence, future water losses are very likely to increase. Nevertheless, our model results indicate a rather moderate increase in water consumption compared to other ski areas in Switzerland and Austria. For Scuol (eastern Alps, CH), the water consumption by the end of the century may rise by a factor of 2.4–5, in Hochjoch (AU) by a factor of 2.2–3.7 (Abegg and Steiger 2016). For the winter season 2007 in the winter tourism region Davos (CH), Rixen et al. (2011) compared the water and energy consumption of the ski resort with the drinking water and energy consumption of the municipality. In terms of energy consumption, the ski resort used less than 1% of the municipality’s energy. But the water use comprised 21.5% of the municipalities drinking water (but using different water sources).

The percental upsurge in water consumption for the region Nätschen/Oberalp, where most of the new ski runs, lifts, and snowmaking facilities were built, is similar as the percental increase for the whole ski area (+ 19% by the mid-century and + 65% by the end of the century, RCP8.5). Most of the water for snowmaking is extracted from the reservoir lake Oberalpsee (max. 200 × 10^3^ m^3^ season^−1^ extraction, pumping rate of 270 L s^−1^). Potential future water resources would be an additional reservoir lake (“Ober Gütsch”, 50 × 10^3^ m^3^ season^−1^) and groundwater in Andermatt (200 × 10^3^ m^3^ season^−1^). With unabated greenhouse gas emissions (RCP8.5 scenario), the reservoir Oberalpsee will suffice for the snowmaking activities of the region until the mid-century, but the water demand will clearly exceed the availability by the end of the century. Additional water resources in the range of 80 × 10^3^ m^3^ without any water losses will be required. Noteworthy, if we reduce the greenhouse gas emissions (RCP4.5), the reservoir of the Oberalpsee will still meet the water demands for snowmaking even by the end of the century. As the other regions’ water sources are rivers, the water availability is much more constrained by interannual fluctuations and there are no defined maximum rates of extraction per year (residual water flows in rivers have to be guaranteed).

### Competition for water

The Oberalpsee is also used for hydroelectric power generation. The power station Oberalp produces three quarters of the energy during snowmelt and the subsequent summer months without snow. The withdrawal of water by the ski resort and by the electrical power company is regulated by law (Elektrizitätswerk Ursern 2020). Hence, competition and conflicts between the hydropower production and snowmaking will arise if the water level of the Oberalpsee and rivers will drop in the future. Such conflicts may emerge mainly in drier regions of Switzerland, such as the Engadine or parts of the Valais. Peaks in water demand for tourism often coincide with generally low water levels (Reynard 2020). Nevertheless, water shortages in Swiss tourism regions are usually caused by unsustainable water management strategies and poor distribution among stakeholders, not by a general lack of water (Clivaz and Reynard 2008; Schneider et al. 2016). A detailed study for the French Isère department showed that the water availability for snowmaking may even increase due to more rain and increased snowmelt, but mainly at catchment areas below 1500 m asl (Gerbaux et al. 2020). In the region of Andermatt, the newly built resort including golf, swimming pools and spa have generated new water demands that have not yet been quantified. We therefore suggest that the increasing water consumption of the ski resort as well as these new sources of water demand is considered in future water management strategies.

In addition to the new demands for water and the climatic changes, there are changes in the land use of the Ursern valley that affect the surface runoff of the catchment. Successive abandoning of extensive grazing on alpine grassland is leading to an expansion of shrubland, in particular of the green alder. Because of the higher evapotranspiration of the green alder and abandoned grassland, this land use change reduces the runoff in the Ursern catchment (Inauen et al. 2013; van den Bergh et al. 2018). However, land use changes primarily affect the runoff during the summer months. Snowmaking usually starts in mid-November or early December and commonly lasts until January. Alaoui et al. (2014) showed that the water discharge of the Ursern valley during these months is clearly dominated by precipitation. As the amount of winter precipitation is expected to shift by − 2 to 24% by the end of the century (NCCS 2018), future competition for water resources in winter will likely not be triggered by a decrease in the supply, but rather by increasing water demands of the ski areas.

## Conclusions

The studied ski resort *Andermatt-Sedrun-Disentis* features a high natural snow reliability throughout the twenty-first century and reductions in natural snow can mainly be compensated by snowmaking. However, under a climate change scenario with unabated emissions, lower areas (below 1800–1900 m asl) as well as the region of Sedrun, even above the critical access elevation, will not be snow-reliable over the Christmas holidays by the end of the twenty-first century, as the climate will not allow for sufficient snow production. Under this scenario, the water consumption will rise by 79% by the end of the century. The currently largest water source, the reservoir lake Oberalpsee, will then not meet the water demands of the region anymore and new sources such as ground water and a new reservoir lake will have to be exploited. According to the climate change scenarios (CH2018), it is likely that the water supply during the months of highest water consumption (November until January) will not decrease, but the high consumption may lead to competition with other sectors such as hydropower or the new hotels. Although the overall demand for skiing tourism in Switzerland has been decreasing since 2008 (SBS 2021), the comparative advantage of *Andermatt-Sedrun-Disentis* (Steiger and Abegg 2018)—in combination with the significant expansion of the resort—will likely lead to an increase in tourist numbers.

### Supplementary Information

Below is the link to the electronic supplementary material.

Supplementary file1 (DOCX 7188 kb)
