# Supplementary material for: Snowmaking in a warmer climate: an in-depth analysis of future water demands for the ski resort Andermatt-Sedrun-Disentis (Switzerland) in the twenty-first century
Source: Int J Biometeorol. 2022 Dec 6;68(3):565–79. doi: 10.1007/s00484-022-02394-z (PMC10864518; doi:10.1007/s00484-022-02394-z)
Supplement: Supplementary file 1 — Supplementary file1 (DOCX 7188 kb) [file 484_2022_2394_MOESM1_ESM.docx]

## **Electronic Supplemental Material (ESM)**


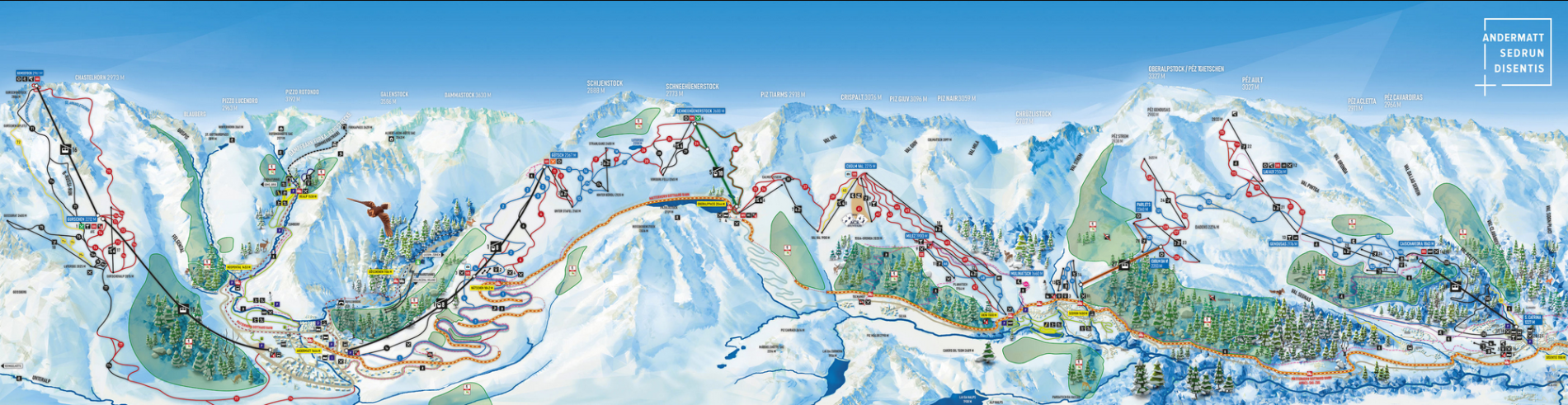


**ESM 1** Map of the ski resort (https://andermatt-sedrun-disentis.ch/; accessed July 22^nd^, 2020)

**ESM 2** The calibrated degree-day factors (in mm water equivalents / K) for the aspect classes and the two weather stations Andermatt and Sedrun

|  | North | | East / west aspect | | South | |
| --- | --- | --- | --- | --- | --- | --- |
|  | min | max | min | max | min | max |
| Andermatt | 0.7 | 3.3 | 1.0 | 5.0 | 1.5 | 7.5 |
| Sedrun | 08 | 3.4 | 1.2 | 5.2 | 1.8 | 7.8 |

**
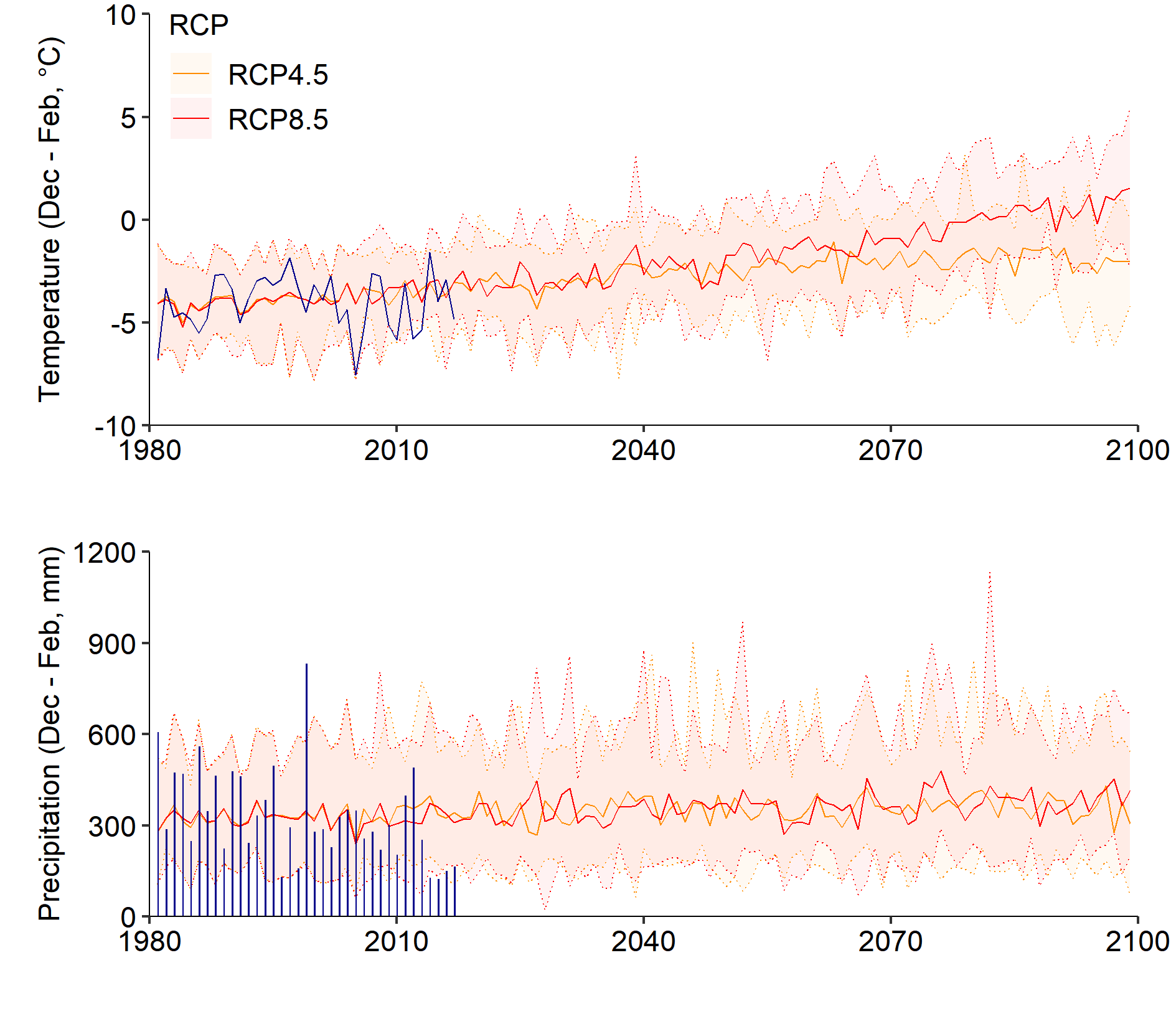
 ESM 3** Mean winter temperature and the winter precipitation sum (December to February) at the weather station Andermatt (1442 m asl). Measured temperature and precipitation are shown in dark blue (Federal Office of Meteorology and Climatology MeteoSwiss, (1981-2017, period refers to the start of the reference period of the climate change scenarios until last season's data of water usage for snowmaking). The mean, minimum and maximum of all simulations of the Swiss climate change scenarios (NCCS, 2018) are depicted in orange (RCP4.5) and red (RCP8.5, 1981-2099)

**
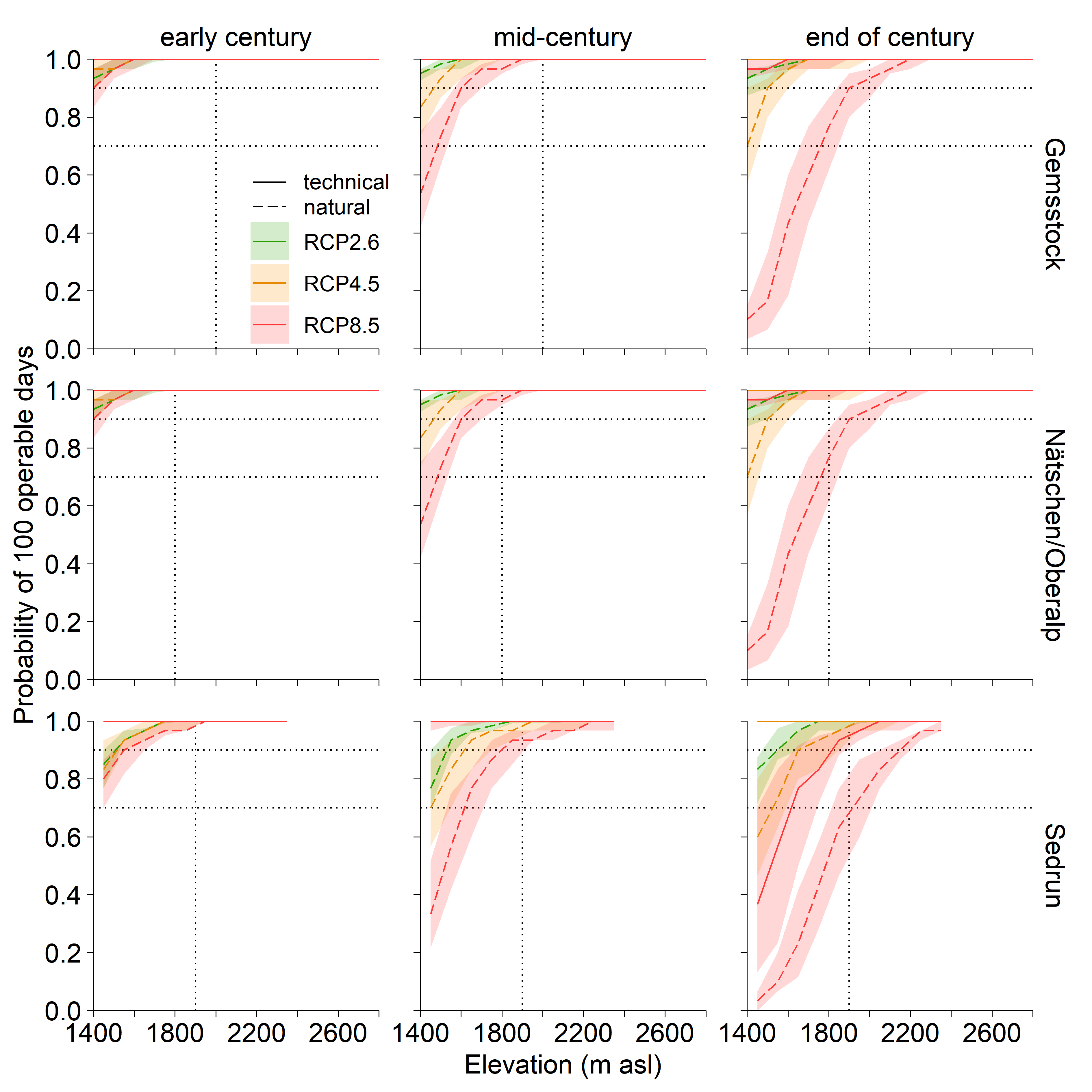
ESM 4** The probability of **100 consecutive days** that are operable for skiing on natural snow (dashed line) and with technical snow (solid line) for the **north**-exposed slopes of the three regions Gemsstock, Nätschen/Oberalp, Sedrun under the three RCP scenarios and for three time periods of the 21^st^ century. The lines represent the median of all simulations per RCP scenario and 50% of the simulations lie in the shaded ribbon. The horizontal lines indicate the snow reliability at 70% and 90%, respectively, the vertical lines the critical access elevation. At a probability of 1, the lines of the three scenarios overlap

**
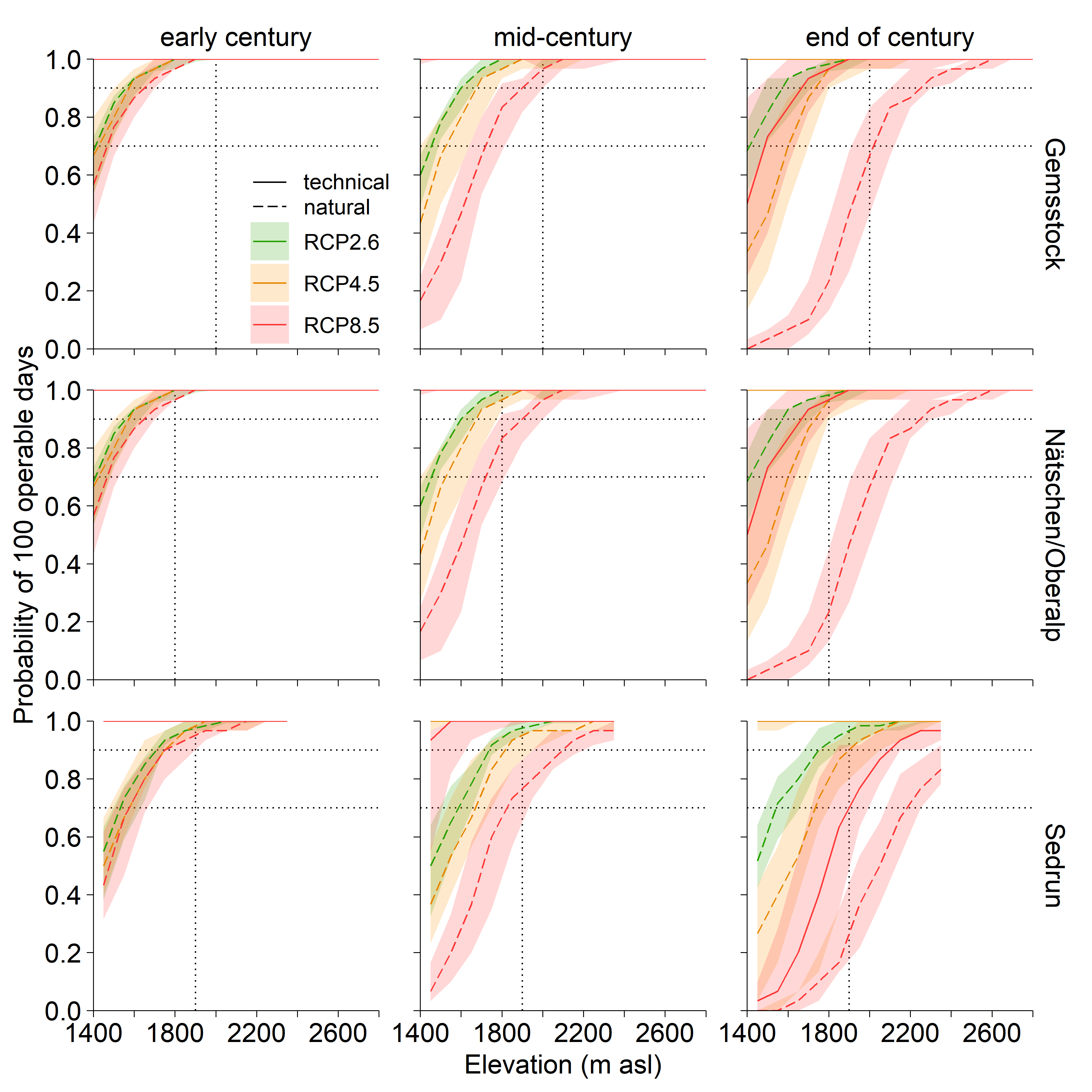
ESM 5** The probability of **100 consecutive days** that are operable for skiing on natural snow (dashed line) and with technical snow (solid line) for the **south**-exposed slopes of the three regions Gemsstock, Nätschen/Oberalp, Sedrun under the three RCP scenarios and for three time periods of the 21^st^ century. The lines represent the median of all simulations per RCP scenario and 50% of the simulations lie in the shaded ribbon. The horizontal lines indicate the snow reliability at 70% and 90%, respectively, the vertical lines the critical access elevation. At a probability of 1, the lines of the three scenarios overlap

**
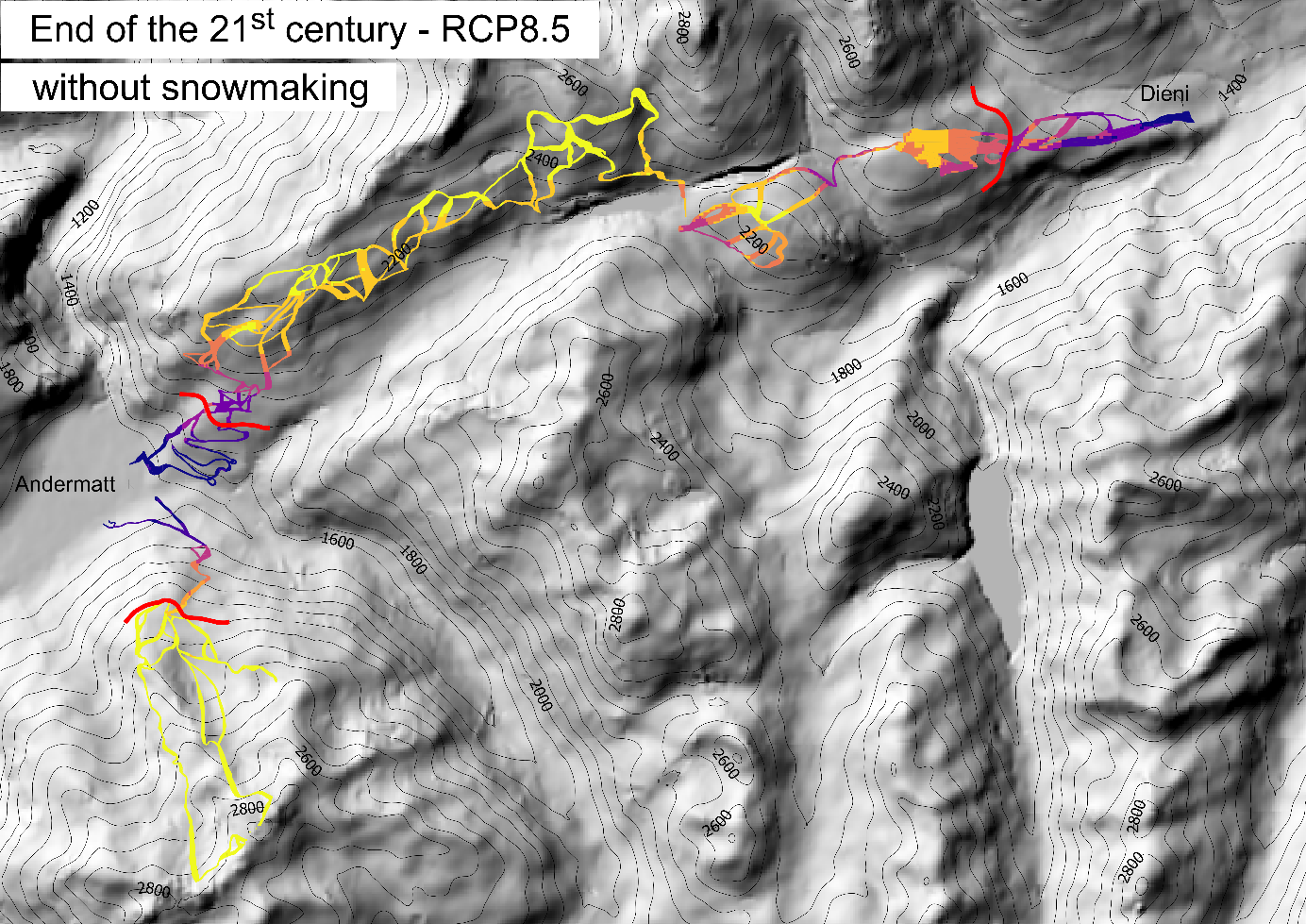

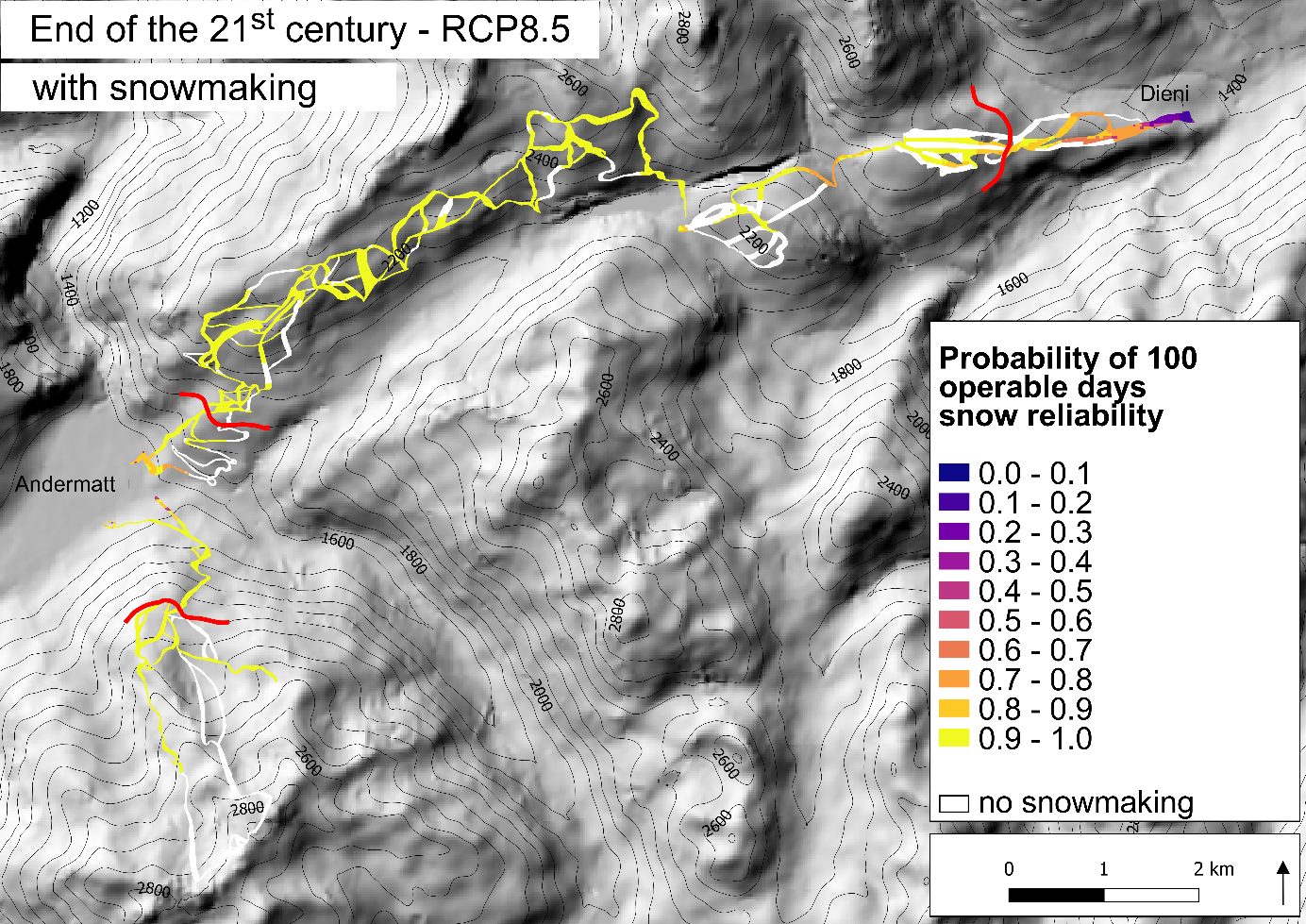
ESM 6** The probability of 100 operable days with and without snowmaking at the end of the century under RCP8.5. Areas depicted in white are not serviceable for snowmaking and the red line is the critical access elevation

**
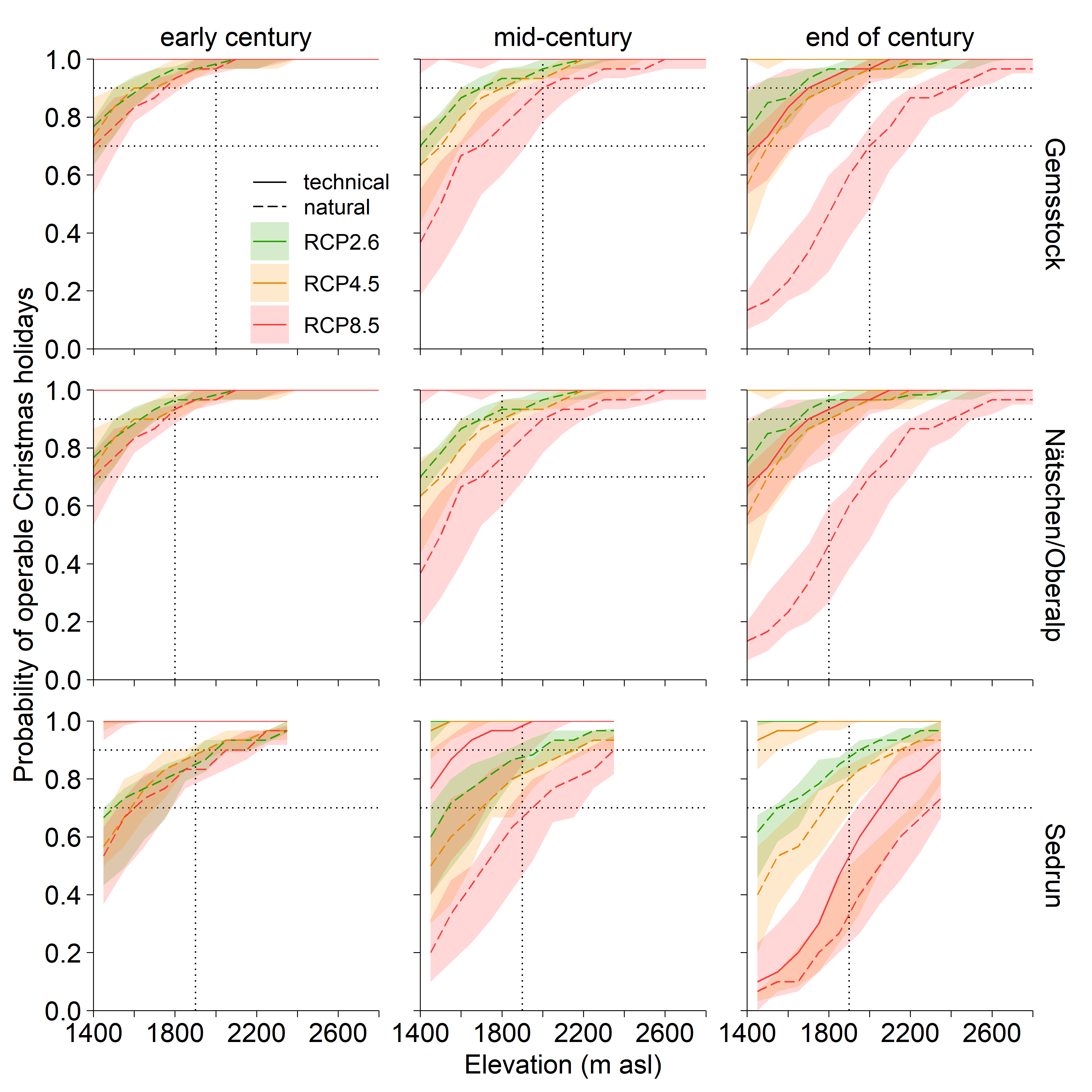
ESM 7** The probability that the resort is operable over the **Christmas holidays** on technical snow (solid line) and with natural snow only (dashed line) for the **north**-exposed slopes of the three regions Gemsstock, Nätschen/Oberalp, Sedrun under the three RCP scenarios and for three time periods of the 21^st^ century. The lines represent the median of all simulations per RCP scenario and 50% of the simulations lie in the shaded ribbon. The horizontal lines indicate the snow reliability at 70% and 90%, respectively, the vertical lines the critical access elevation. At a probability of 1, the lines of the three scenarios overlap

**
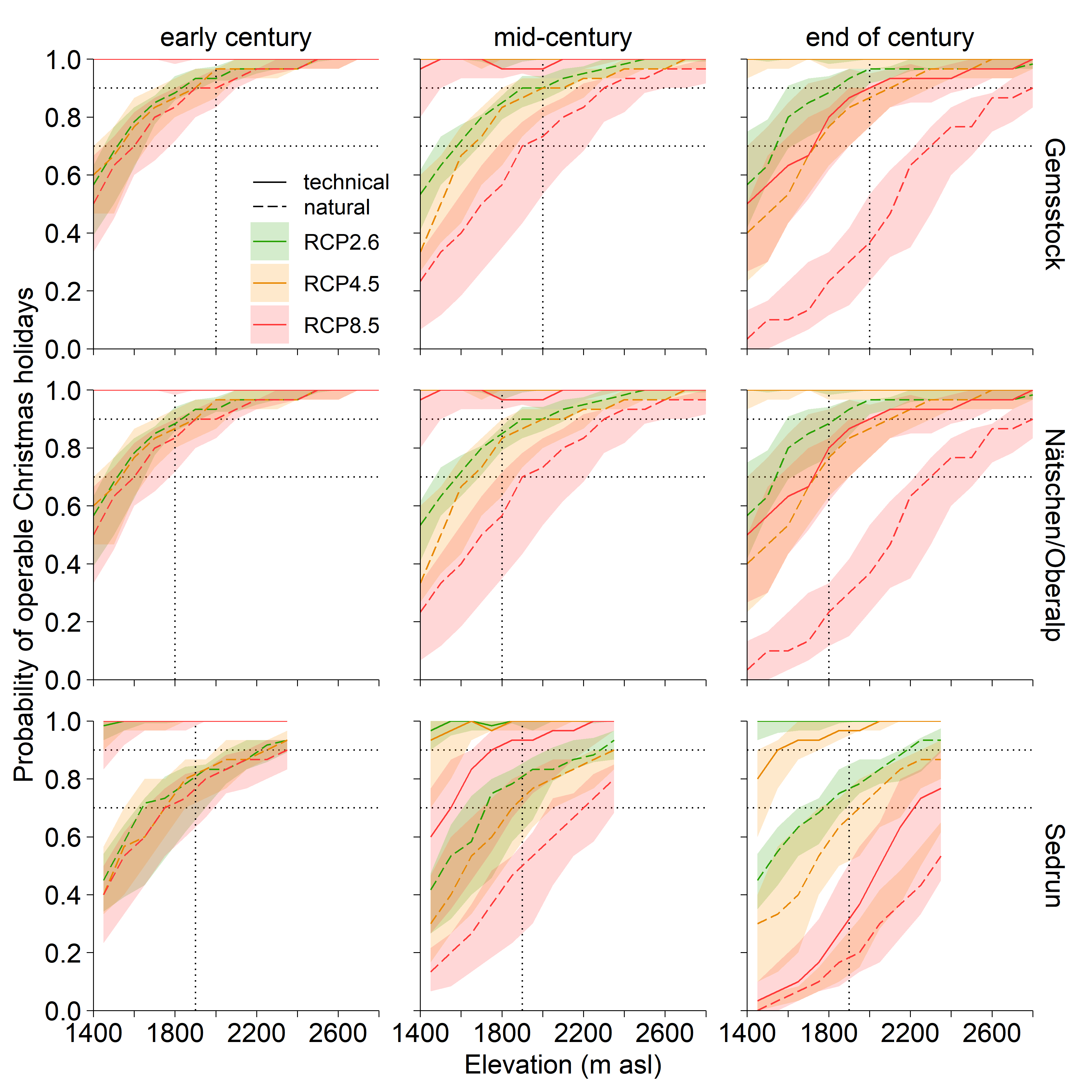
 ESM 8** The probability that the resort is operable over the **Christmas holidays** on technical snow (solid line) and with natural snow only (dashed line) for the **south**-exposed slopes of the three regions Gemsstock, Nätschen/Oberalp, Sedrun under the three RCP scenarios and for three time periods of the 21^st^ century. The lines represent the median of all simulations per RCP scenario and 50% of the simulations lie in the shaded ribbon. The horizontal lines indicate the snow reliability at 70% and 90%, respectively, the vertical lines the critical access elevation. At a probability of 1, the lines of the three scenarios overlap
